# The Role of Prostaglandin-Endoperoxide Synthase-2 in Chemoresistance of Non-Small Cell Lung Cancer

**DOI:** 10.3389/fphar.2019.00836

**Published:** 2019-08-08

**Authors:** Xiao-mian Lin, Wu Luo, Heng Wang, Rong-zhen Li, Yi-shan Huang, Lian-kuai Chen, Xiao-ping Wu

**Affiliations:** Institute of Tissue Transplantation and Immunology, Jinan University, Guangzhou, China

**Keywords:** chemoresistance, cisplatin, non-small cell lung cancer (NSCLC), prostaglandin-endoperoxide synthase-2 (PTGS2), therapeutic target

## Abstract

The prostaglandin-endoperoxide synthase-2 (PTGS2) plays essential roles in diverse pathological process. Although recent studies implied that PTGS2 was closely related with chemoresistance, the precise roles and the underlying mechanisms of PTGS2 in the developing process of chemoresistance in non-small cell lung cancer (NSCLC) remained elusive. In the present study, we revealed a novel molecular mechanism of PTGS2 implicated in the chemoresistance of NSCLC and proposed a model for the positive feedback regulation of PTGS2 in the process of developing resistance phenotype in NSCLC cells. Our results demonstrated that cisplatin induced PTGS2 expression through the ROS-ERK1/2-NF-κB signaling axis. The prostaglandin E2 (PGE2) derived from PTGS2 catalyzation further strengthened PTGS2 expression *via* the PGE2-EPs-ERK1/2 positive feedback loop, which induced multidrug resistance of NSCLC cells through up-regulation of BCL2 expression and the subsequent attenuation of cell apoptosis. Consistently, high levels of both PTGS2 and BCL2 were closely associated with poor survival in NSCLC patients. Inhibition of PTGS2 significantly reversed the chemoresistance in the resistant NSCLC *in vitro* and *in vivo*. Our results suggested that PTGS2 might be employed as an adjunctive therapeutic target for improving the response to the therapeutic agents in a subset of resistant NSCLC.

## Introduction

Lung cancer, especially non-small cell lung cancer (NSCLC) (Liu et al., 2018), is a common malignant tumor that seriously damages human health and life (Ferlay et al., 2015; Chen et al., 2016; Shen et al., 2017). It is also the most malignant tumor with the highest morbidity and mortality worldwide. At present, platinum-based chemotherapy is the most important standard chemotherapy regimen for lung cancer (Azzoli et al., 2010; Reck et al., 2014). However, the acquisition of the acquired multidrug resistance is a major cause for the failure of chemotherapy (Beretta et al., 2004; Wu et al., 2005; Chen et al., 2008; Gu et al., 2009; Wang et al., 2009; Biagosch et al., 2010; Lavi et al., 2012; Wang et al., 2013; Li et al., 2014). Cisplatin is an effective first-generation platinum agent widely used in NSCLC treatment (Han et al., 2008). Mechanisms including reduction of drug uptake, enhancement of DNA repair, and detoxification efficacy have been proposed to be responsible for the acquired resistance to cisplatin-based chemotherapy (Galluzzi et al., 2014). Further clarifying the molecular mechanism of cisplatin resistance is essential to design therapeutic regimen for improving efficacy of cisplatin in NSCLC.

PTGS2 is an inducible enzyme with potential of catalyzing arachidonic acid to prostaglandins, which exert diverse physiological and pathological functions (Liu et al., 2015b; Rumzhum and Ammit, 2016). Overproduction of PTGS2 and the prostaglandins was observed in lung cancer and involved in lung carcinogenesis and progression *via* promoting angiogenesis, metastasis, and immunosuppression (Huang et al., 1998; Hida et al., 2000; Dohadwala et al., 2001; Mao et al., 2003). Although recent reports showed that PTGS2 was also implicated in chemoresistance of certain malignant tumors including liver, pancreatic, bladder, esophageal, and gastric cancers (Gee et al., 2006; Zhi et al., 2006; Harris, 2009; Mukherjee et al., 2009; Wang and Dubois, 2010; Wang et al., 2014; Liu et al., 2015a; Liu et al., 2017), and inhibition of PTGS2 effectively enhanced the sensitivity of tumors to drugs, the detailed mechanisms by which PTGS2 contributing to the development of chemoresistance remained elusive. In the present study, we revealed a novel molecular mechanism of PTGS2 mediating BCL2 expression and the subsequent apoptosis and resistance, and proposed a model for the positive feedback regulation of PTGS2 in the process of developing resistance phenotype in NSCLC cells. In addition, we also demonstrated that targeting PTGS2 might be an effective strategy for improving the sensitivity to chemotherapy in a subset of cisplatin-resistant NSCLC.

## Materials and Methods

### Materials

#### Reagents

TRIzol^®^ was from Thermo Fisher Scientific, Inc. (Waltham, MA, USA). First-strand cDNA synthesis kit and SYBR green q-PCR kit were purchased from Bio-Rad Laboratories, Inc. (Hercules, CA, USA). Primary antibodies against the following proteins were purchased from Cell Signaling Technology, Inc. (Danvers, MA, USA): PTGS2 mAb (cat. no. 12282S), BCL2 mAb (cat. no. 13110), GAPDH mAb (cat. no. 8884), ERK mAb (cat. no. 4695S), p-ERK mAb (cat. no. 4370S), PCNA mAb (cat. no. 2586T), and NF-κB mAb (cat. no. 8242). Cisplatin (DDP) was from Qilu-pharma Inc. (Shandong, China), doxorubicin (Adriamycin, ADM) was from Shanghai Bioengineering Co., Ltd (Shanghai, China), and Gemcitabine (GEM) was from Aladdin (Shanghai, China). Annexin V-APC/7-ADD apoptosis kit (KA3809) was the product of Abnova (Taiwan, China). PTGS2 inhibitor (celecoxib) and EPs inhibitor (L-161982) were obtained from Selleck Chemicals (Houston, Texas, United States). ERK inhibitor (U0126) was the product of Cell Signaling Technology, Inc. (Danvers, MA, USA). ROS assay kit (S0033-1) was from Beyotime Biotechnology (Shanghai, China). PVDF membrane and ECL detection kit were from Millipore (Billerica, MA, USA). Enzyme-linked immunosorbent assay (ELISA) kit of PGE2 (E-EL0034c) was the product of Elabscience Biotechnology Co. Ltd (Wuhan, Hubei, China).

#### Cell Culture

H460 cell line was purchased from *SGST.CN* (Shanghai, China) and cultured in RPMI-1640 media containing 10% FBS. HEK-293T cell line was kept by the Institute of Tissue Transplantation and Immunology and cultured in DMEM media supplemented with 10% FBS. A549 and A549/DDP cell lines were kindly provided by Professor Meng Xu in the Faculty of Medical Science of Jinan University. A549 cells were cultured in RPMI-1640 media plus 10% FBS, while A549/DDP cells were in RPMI-1640 media containing 10% FBS and 500 ng/ml DDP.

#### Animals

Four-week-old male BALB/c nude mice (*n* = 31) were purchased from Beijing Hua-Fu-Kang Company and prepared to establish models after isolation for 1 week. Animal experiments carried out in the Laboratory Animal Institute were under the supervision and assessment by the Laboratory Animal Ethics Committee of Jinan University.

### Methods

#### Database

Gene expression profiles were obtained from GEO Profiles (https://www.ncbi.nlm.nih.gov/geoprofiles). Survival Curves were generated by Kaplan–Meier plotter (http://kmplot.com/analysis/index.php).

#### Cell Viability Assay

Cells were seeded in 96-well plates and attached overnight, and then treated with DDP, ADM, or GEM for 48 h. For groups of drug combination, cells were incubated with celecoxib for 6 h prior to addition of DDP, ADM, or GEM. The cell viability was determined by the methylthiazole tetrazolium (MTT) colorimetric assay. After incubation with MTT for 4 h, plates were immediately measured at the wavelength of 570 nm using the microplate reader (Bio-Rad, Hercules, CA, USA). The IC_50_ values were determined from dose–response curves.

#### Quantitative PCR Analysis

Total RNA was isolated with TRIZOL following the manufacturer’s instructions and subjected to produce cDNA using the first-strand cDNA synthesis kit, which were then served as templates for quantitative PCR (q-PCR) amplification with the SYBR green q-PCR kit. The primers are shown in **Table S1**. The PCR conditions were 94°C for 5 min followed by 40 cycles of 95°C for 5 s, 58°C–59°C for 10 s, and 72°C for 20 s. GAPDH was amplified as an internal control.

#### Flow Cytometry Analysis

Cells were seeded in plates and attached overnight. For groups of co-treatment, cells were treated with celecoxib for 6 h prior to addition of cisplatin. After 48 h, cells were collected and stained with annexin-V/APC and 7-AAD for 15 min followed by flow cytometry analysis.

#### Construction of Stable Cell Lines Overexpressing or Knocking Down PTGS2

In brief, PTGS2 gene fragment amplified from A549/DDP cDNA was digested with *EcoRI* and *BamHI*, and ligated with the vector pCDH513B digested with the identical enzymes to obtain the recombinant plasmid pCDH513B/PTGS2 (pCDH/PTGS2). Meanwhile, the annealing oligonucleotides were ligated with GV248 vector digested with *EcoRI* and *AgeI* to construct the recombinant plasmid GV248/shPTGS2. Primers and oligonucleotides (**Table S1**) were synthesized by Shanghai Bioengineering Co., Ltd. (Shanghai, China). The recombinant plasmids pCDH/PTGS2 and GV248/shPTGS2 were co-transfected with psPAX2 and pMD2.G into HEK-293T cells to package the lentivirus, which were used to infect cells to obtain the stable cell lines overexpressing or knocking down PTGS2 by puromycin screening.

#### Western Blot Analysis

The protein samples were subjected to run onto 10% SDS-PAGE and transferred to a PVDF membrane, which was blocked at room temperature for 1 h in TBST containing 5% non-fat dry milk, and subsequently incubated with various primary antibodies at 4°C overnight. After incubation with goat anti-rabbit HRP-linked antibody for 1 h at room temperature, the blots were visualized using an ECL detection kit and analyzed by Quantity One software to determine the ratio relative to GAPDH or PCNA.

#### PGE2 Secretion Assay by ELISA

The levels of prostaglandin E2 (PGE2) were determined using ELISA kits (Elabscience, Wuhan, Hubei, China) following the manufacturer’s procedure. Briefly, the cultured media were collected and immediately incubated with the biotinylated antibody working solution at 37°C for 45 min, followed by treatment with enzyme working solution at 37°C for 30 min. HRP substrate solution (TMB) was added to each well and incubated at 37°C for 15–30 min prior to termination. The OD value was immediately measured at a wavelength of 450 nm using the microplate reader.

#### Reactive Oxygen Species Detection

Cells were treated with NAC for 6 h to scavenge reactive oxygen species (ROS) prior to administration of cisplatin. The intracellular ROS levels were detected by DCFH-DA assay. Briefly, cells were incubated with 2',7'-Dichlorodihydrofluorescein diacetate (DCFH-DA) (5 μM) for 20 min at 37°C in the dark, washed three times with serum-free medium, and subjected to flow cytometric assay. FCS Express Version 3 software and FlowJo 7.6.1 were used to determine the ROS levels.

#### PTGS2 Promoter Activity Reporter Assay

The EGFP reporter plasmid pGL3-EGFP was constructed as previously described (Luo et al., 2016). The PTGS2 promoter region (−1,047 to +1) was amplified from human genomic DNA extracted from HUVEC cells using a primer pair, and inserted into pGL3-EGFP vector between *XhoI* and *Hind III* to construct a PTGS2 promoter/EGFP reporter plasmid pGL3-proPTGS2-EGFP. For promoter activity reporter assay, cells were co-transfected with pGL3-proPTGS2-EGFP and pDsRed1-N1 using Lipofectamine 2000^™^ (Invitrogen, USA). The fluorescence intensity was determined by flow cytometry.

#### *In Vivo* Animal Experiments

The A549 cells (1 × 10^7^) and A5491/DDP cells (5 × 10^6^) were injected into the flank of 5-week-old male BALB/c nude mice, which were randomly distributed into six groups (*n* = 5–6 per group): A549 treated with vehicle (control), A549 treated with cisplatin, A549/DDP treated with vehicle (control), A549/DDP treated with cisplatin, A549/DDP treated with celecoxib, and A549/DDP treated with a combination of cisplatin and celecoxib. When tumors reached around 5 × 5 × 5 mm^3^, celecoxib was intraperitoneally injected at a dose of 5 mg/kg once daily, and the cisplatin was intraperitoneally injected at a dose of 3 mg/kg once every 3 days. The control group received the equivalent volume of PBS buffer alone. Body weights were monitored every 3 days. After scarification, tumors and organs were dissected and weighed. The tumor inhibition rate was calculated using the formula:

$$\begin{array}{l} \text{Inhibition rate (\%)}=\\ \left(1-\frac{\mathrm{Average tumor weight of the experimental group}}{\mathrm{Average tumor weight of the control group}}\right)\times 100\% \end{array}$$

For immunohistological analysis, tumors were dissected, fixed by 4% paraformaldehyde, and embedded in paraffin prior to probing with anti-PTGS2 and anti-BCL2 antibodies. Images were acquired using an Inverted Fluorescence Microscope (Zeiss, Feldbach, Switzerland) and subjected to analysis of integrated optical density (IOD) and Area by Image-Pro^®^ Plus v 6.0 (for Windows).

#### Statistical Analysis

GraphPad Prism software 5.01 was used for the statistical analyses. Comparisons between two groups were performed by the Student’s *t* test, while multiple comparisons were analyzed by one-way ANOVA followed by Tukey’s multiple-comparison test. Differences were considered significant at *P* < 0.05.

## Results

### PTGS2 Up-Regulated in the Cisplatin-Resistant NSCLC Cells Promoted Multidrug Resistance

**Figure 1:**
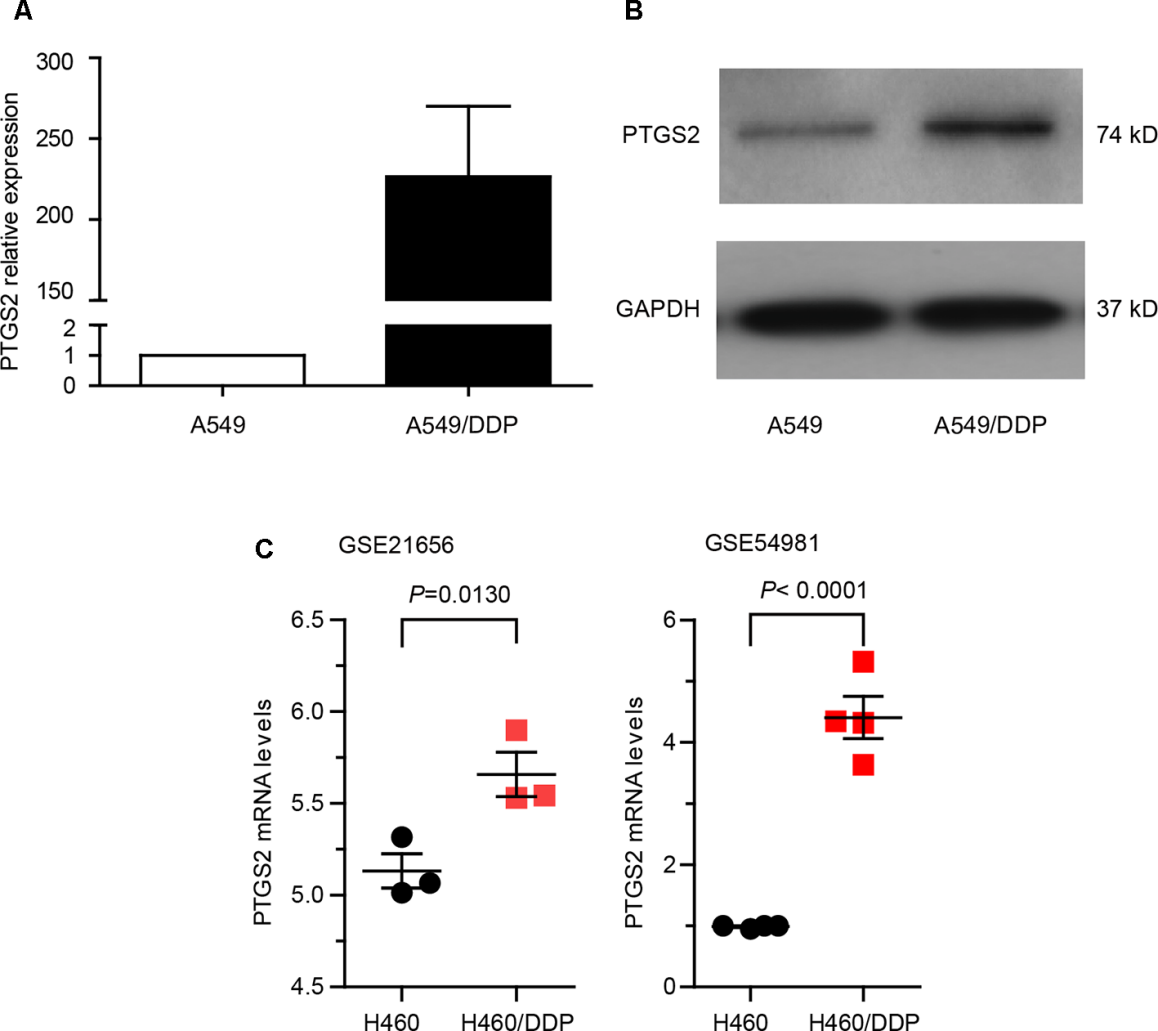
Prostaglandin-endoperoxide synthase-2 (PTGS2) up-regulated in the cisplatin-resistant non-small cell lung cancer (NSCLC) cells promoted multidrug resistance. **(A** and **B)** Comparison of PTGS2 expression in A549 and A549/DDP cells were carried out by quantitative PCR (q-PCR) and Western blotting. A549 and A549/DDP cells (5 × 10^6^) were lysed to obtain samples for Reverse Transcription - quantitative Polymerase Chain Reaction (RT-qPCR) and Western blot analysis. Fold change is calculated relative to A549 cells. **(C)** Analysis of two datasets (GSE21656 and GSE54981) in GEO (Gene Expression Omnibus). Data analysis indicated that PTGS2 was elevated in H460/DDP cells.

In order to explore whether PTGS2 is involved in the chemoresistance of NSCLC, we first compared the expression levels of PTGS2 between the NSCLC cell line A549 and the corresponding cisplatin-resistant cell line A549/DDP, which displayed significant higher resistance to cisplatin (DDP), adriamycin (ADM), and gemcitabine (GEM) than A549 cells (**Table S2**). As shown in **Figure 1 (A–B)** and **Data Sheet S1**, remarkably elevated mRNA and protein levels of PTGS2 were observed in A549/DDP cells. Analysis of two datasets (GSE21656 and GSE54981) in GEO (Gene Expression Omnibus) also indicated that the resistant cell line H460/DDP expressed higher levels of PTGS2 than the parental H460 cells (**Figure 1C**). The results suggested that PTGS2 up-regulated in the resistant cells might play essential roles in the chemoresistance of NSCLC cells.

To further clarify the roles of PTGS2 in the chemoresistance of NSCLC cells, stable cell lines overexpressing or knocking down PTGS2 (A549-pCDH/PTGS2, H460-pCDH/PTGS2, or A549/DDP-GV248/shPTGS2) were constructed (**Figure S1** and **Data Sheet S2**). The effects of alteration of PTGS2 expression on the chemoresistance were assessed by the MTT method. The results showed that overexpression of PTGS2 increased the resistance to DDP, ADM, and GEM, with higher IC_50_ values of A549-pCDH/PTGS2 and H460-pCDH/PTGS2 cells than those of the corresponding A549-pCDH and H460-pCDH cells transfected with the control vector (**Table 1A**). Knocking down of PTGS2 decreased the resistance to the chemical agents evidenced by the lower IC_50_ value detected in A549/DDP-GV248/shPTGS2 compared with A549/DDP-GV248 cells (**Table 1B**). Inhibition of PTGS2 activity with celecoxib in A549/DDP cells also led to enhanced sensitivity of the resistant cells against DDP, ADM, and GEM (**Table 1B**). The results confirmed that PTGS2 contributed to the chemoresistance of NSCLC cells.

**Table 1:** Prostaglandin-endoperoxide synthase-2 (PTGS2) promoted multidrug resistance in non-small cell lung cancer (NSCLC) cells.

(A)				
Cells	Groups	IC_50_		
		DDP (µg/ml)	ADM (µg/ml)	GEM (µg/ml)
A549	pCDH	1.3 ± 0.02	1.3 ± 0.11	0.8 ± 0.13
	pCDH/PTGS2	4.5 ± 0.41	3.7 ± 0.33	7 ± 0.40
H460	pCDH	0.5 ± 0.01	0.9 ± 0.08	2.0 ± 0.15
	pCDH/PTGS2	4.2 ± 0.41	4.7 ± 0.35	12.0 ± 0.40
(B)				
Cells	Groups	IC_50_		
		DDP (µg/ml)	ADM (µg/ml)	GEM (µg/ml)
A549/DDP	GV248	4.5 ± 0.33	4.6 ± 0.45	8.2 ± 0.56
	GV248/shPTGS2	1.5 ± 0.02	1.1 ± 0.08	2.1 ± 0.33
A549/DDP	DMSO	4.2 ± 0.32	4.3 ± 0.41	8.1 ± 0.49
	Celecoxib (30 µM)	1.6 ± 0.05	1.3 ± 0.09	2.3 ± 0.32

### PTGS2 Enhanced the Chemoresistance *via* Suppression of Cisplatin-Triggered Apoptosis in NSCLC Cells

**Figure 2:**
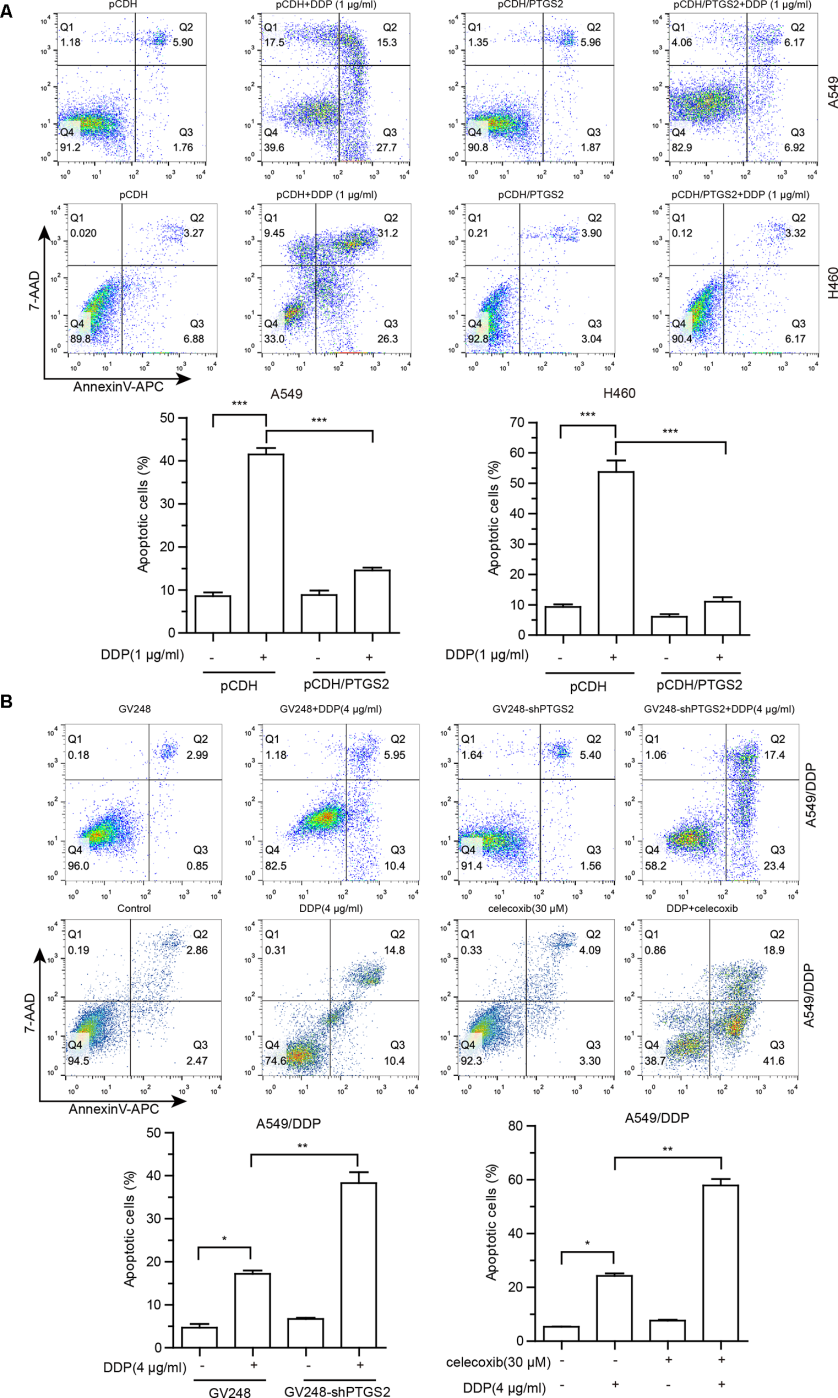
The effects of PTGS2 on cisplatin-induced apoptosis. **(A** and **B)** Cells were plated at a density of 2.5 × 10^5^/well in 12-well plates. After 12 h, cells were treated with 1 µg/ml or 4 µg/ml cisplatin for 48 h. For groups of co-treatment, cells were pre-treated with celecoxib for 6 h prior to the treatment of cisplatin. Cell apoptosis was quantified by annexin-V/APC and 7-AAD staining. *P < 0.05; **P < 0.01; ***P < 0.001.

Cisplatin exerts antitumor effects *via* inducement of cell apoptosis (Cohen and Lippard, 2001; Mandic et al., 2003; Kelland, 2007), and impairment of the apoptosis is an important mechanism underlying the development of chemoresistance. To investigate the effects of PTGS2 on cisplatin-induced apoptosis, flow cytometry analysis combined with Annexin V-APC and 7-AAD double staining was performed. As shown in **Figure 2**, overexpression of PTGS2 significantly decreased cisplatin-induced apoptosis from 43.00% to 13.09% in A549 cells and from 57.50% to 9.49% in H460 cells, respectively. In contrast, PTGS2 knockdown increased the apoptotic rate from 16.35% to 40.8%, and inhibition of PTGS2 activity also elevated the apoptotic rate from 25.2% to 60.5% in A549/DDP cells. Collectively, the results suggested that PTGS2 might enhance the resistance of NSCLC cells through suppression of cell apoptosis triggered by cisplatin.

### PTGS2 Up-Regulated BCL2 Expression

**Figure 3:**
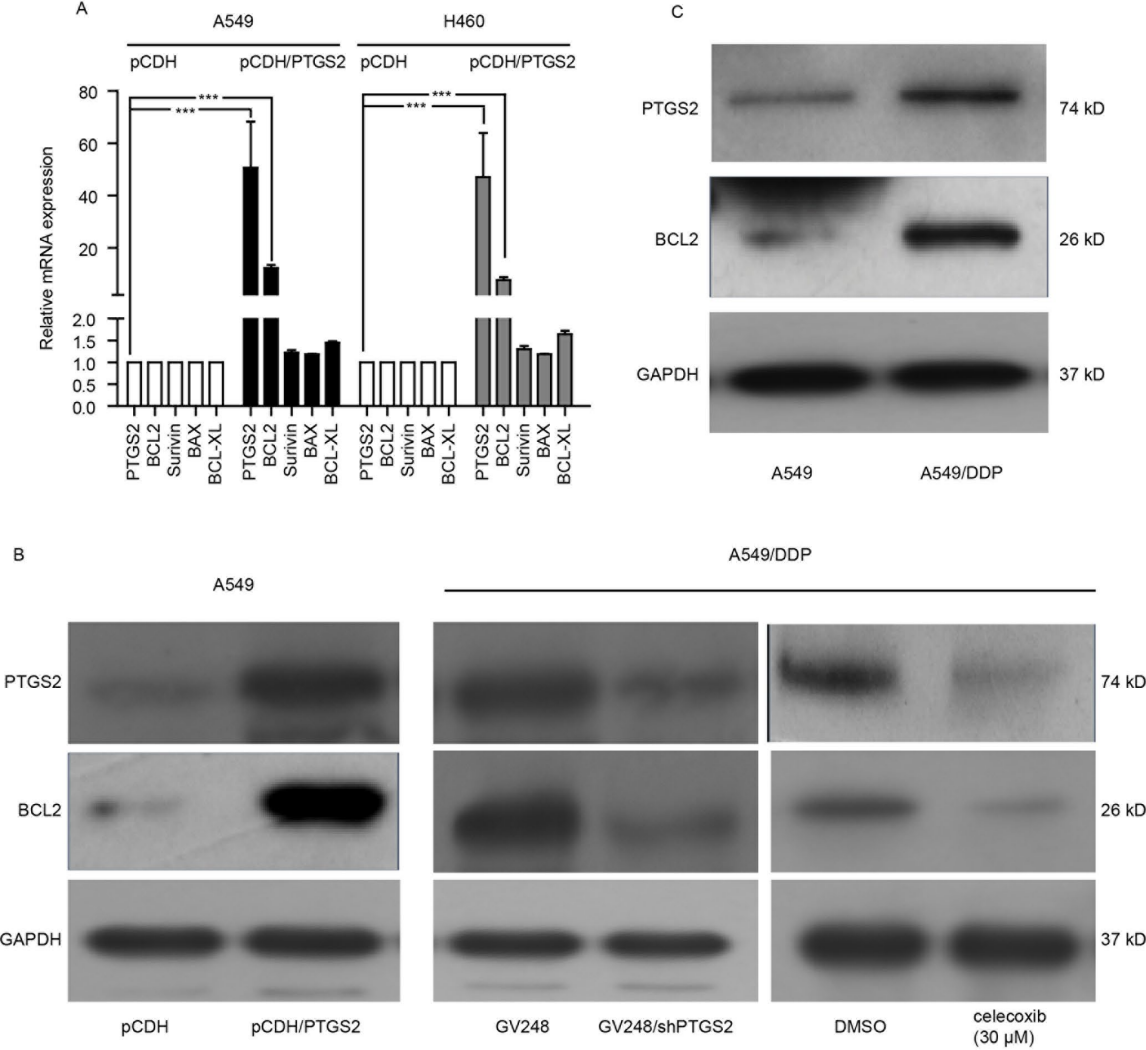
PTGS2 up-regulated BCL2 expression. **(A)** Analysis of PTGS2 on the expressions of apoptosis-related genes (*BCL2*, *Survivin*, *BAX*, and *BCL-XL*) in NSCLC cells by q-PCR. Cells (5 × 10^6^) were lysed by Trizol to obtain total RNA, which was subjected to produce cDNA for q-PCR analysis of the apoptosis-related genes. **(B)** A549-pCDH and A549-pCDH/PTGS2 cells (2.5 × 10^6^) or A549^/^DDP-GV248 and A549^/^DDP-GV248/shPTGS2 cells (3 × 10^6^) were lysed to obtain protein samples for immunoblot analysis with PTGS2 and BCL2 antibodies. **(C)** Comparison of PTGS2 and BCL2 expressions in A549 and A549/DDP cells by Western blotting. ***P < 0.001.

To further investigate the mechanisms of PTGS2 regulating the apoptosis, we detected the effects of PTGS2 on the expressions of the classical apoptosis-related proteins. The results of the quantitative PCR showed that overexpression of PTGS2 enhanced *BCL2* gene expression in both A549 and H460 cells, whereas no significant change of other apoptosis-related genes (*Survivin*, *BAX*, and *BCL-XL*) was observed (**Figure 3A**), which was confirmed by Western blot analysis in **Figure 3B**. In contrast, knocking down or inhibition of PTGS2 in A549/DDP cells led to a decrease of BCL2 expression detected by Western blot analysis (**Figure 3B** and **Data Sheet S3A**). In addition, compared with A549 cells, both PTGS2 and BCL2 were up-regulated in the resistant A549/DDP cells (**Figure 3C** and **Data Sheet S3B**). The results implied that PTGS2 might inhibit cell apoptosis *via* increasing the expression of the anti-apoptotic protein BCL2, which enhanced the resistance of NSCLC cells against the cisplatin.

### Cisplatin Induced PTGS2 *via* an ROS-Mediated ERK1/2-NF-κB Pathway

**Figure 4:**
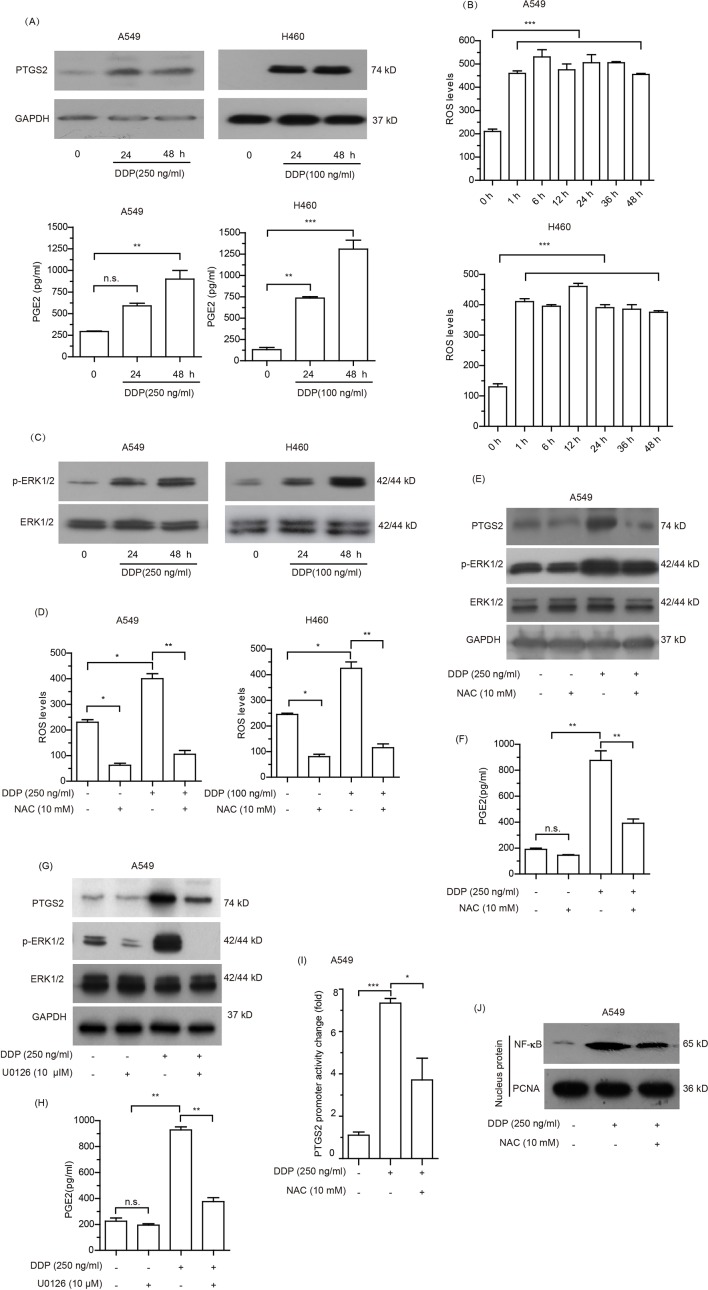
Cisplatin induced PTGS2 *via* reactive oxygen species (ROS) mediated ERK1/2-NFκB pathway. **(A)** Cells were treated with cisplatin and subjected to analysis of PTGS2 expression by Western blotting, as well as PGE2 generation by ELISA as described in the Materials and Methods. **(B)** Cells seeded in 6-well plates were treated with cisplatin for the indicated times, and subjected to detection of the levels of reactive oxygen species (ROS) by flow cytometry. Values were expressed as the means ± SEM of three separate experiments. **(C)** Cells were plated at a density of 5 × 10^5^/well in 6-well plates and treated with cisplatin for 24 and 48 h prior to immunoblot analysis of ERK1/2 activation. **(D**–**F)** Cells were treated with 10 mM NAC for 6 h before stimulation with cisplatin, and subjected to detection of the levels of reactive oxygen species (ROS) by flow cytometry, expressions of PTGS2 and the activation of ERK1/2 by Western blot analysis, and PGE2 generation by ELISA. **(G** and **H)** A549 cells were treated with 10 µM U0126 for 12 h before stimulation with 250 ng/ml cisplatin, followed by detection of PTGS2 expression and ERK1/2 activation by Western blotting, as well as PGE2 generation by ELISA. **(I** and **J)** Cells were treated with 10 mM NAC for 6 h prior to stimulation with 250 ng/ml cisplatin and subjected to detection of the PTGS2 promoter activity and NF-κB translocation by flow cytometry and Western blot analysis, respectively. *P < 0.05, **P < 0.01, ***P < 0.001 and “n.s.” means “no significance.” The loading of each compartment in immunoblot analysis was indicated by GAPDH (cytoplasmic) and PCNA (nucleus).

Given that PTGS2 is an enzyme induced by external factors, we speculated that PTGS2 might be induced in NSCLC cells exposed to the cisplatin. Therefore, we further explored the effects and the underlying mechanisms of cisplatin treatment on PTGS2 expression. As shown in **Figure 4A** and **Data Sheet S4A**, elevated levels of PTGS2, as well as PGE2, the major product catalyzed by PTGS2, were observed in both A549 and H460 cells exposed to cisplatin treatment. Further investigation revealed that the high levels of the intracellular reactive oxygen species (ROS) were detected after treatment with cisplatin for 1 h and maintained for at least 48 h (**Figure 4B**). Analysis of the effects of cisplatin on the activations of MAPK and AKT signal cascades indicated that only the ERK1/2 signal pathway was significantly stimulated by cisplatin (**Figure 4C** and **Data Sheet S4B**). Scavenging of ROS by *N*-acetyl-cysteine (NAC) (**Figure 4D**) significantly inhibited the cisplatin-induced PTGS2 expression as well as activation of the ERK1/2 axis (**Figure 4E** and **Data Sheet S4C**) and PGE2 product (**Figure 4F**), which were also down-regulated by the inhibitor of ERK1/2 (U0126) (**Figures 4G, H** and **Data Sheet S4D**). In addition, administration of cisplatin remarkably promoted the promoter activity of PTGS2 gene and the translocation of nuclear factor-kappa B (NF-κB), an essential transcriptional factor of the PTGS2 gene, both of which were also inhibited by scavenging of ROS (**Figures 4I, J** and **Data Sheet S4E**). Taken together, the results suggested that the cisplatin might induce PTGS2 expression through ROS-mediated activation of ERK1/2 cascade and the subsequent promotion of NF-κB translocation and PTGS2 gene transcription.

### PGE2 Strengthened PTGS2 Expression Through the PGE2-EPs-ERK1/2 Positive Feedback Loop

**Figure 5:**
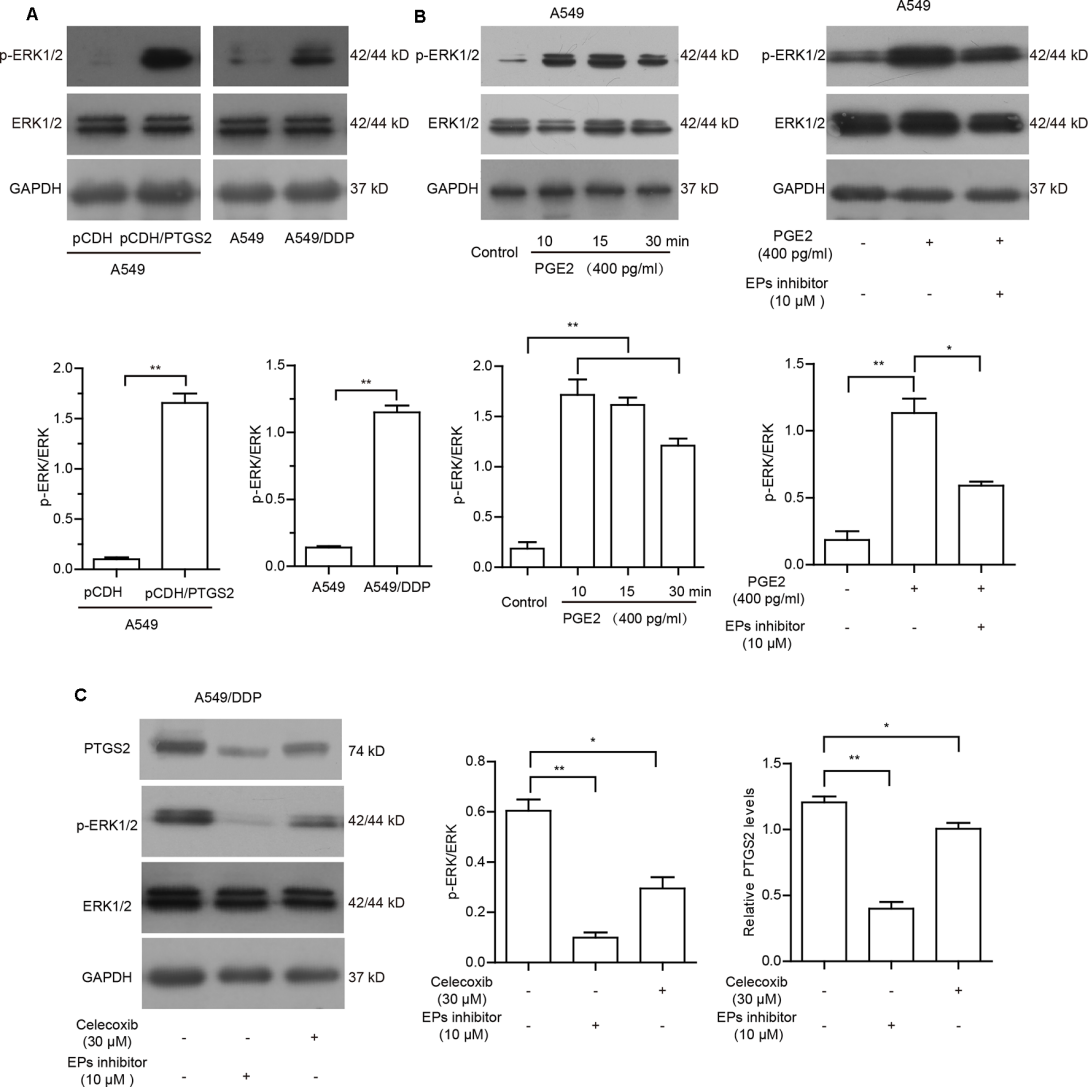
PGE2 strengthened PTGS2 expression through the PGE2-EPs-ERK1/2 positive feedback loop. **(A)** A549-pCDH and A549-pCDH/PTGS2 cells (2.5 × 10^6^), or A549 and A549^/^DDP cells (3 × 10^6^) were lysed to obtain protein samples for Western blot analysis of ERK1/2 activation. **(B)** A549 cells were treated with 400 pg/ml PGE2 for 10, 15, and 30 min, or treated with 10 µM EPs inhibitor for 1 h before addition of 400 pg/ml PGE2, followed by Western blot analysis of ERK1/2 activation. **(C)** A549/DDP cells were treated with 30 µM celecoxib or 10 µM EPs inhibitor prior to Western blot analysis of PTGS2 expression and ERK1/2 activation.

To further clarify whether cisplatin-induced PTGS2 and PGE2 product influenced PTGS2 expression in a positive feedback manner, we first detected the effects of PTGS2 on the activation of ERK1/2 cascade. As shown in **Figure 5A** and **Data Sheet S5A**, overexpression of PTGS2 could enhance the phosphorylation level of ERK1/2, which was also found in the activation state in the resistant A549/DDP cells. Since PGE2 exerts the bioactivities *via* activations of downstream signaling pathways followed by binding to the specific G protein coupled receptor EPs (EP1–4), we further determined whether EPs-ERK1/2 axis contributed to PGE2-mediated PTGS2 expression. The results indicated that administration of PGE2 triggered the activation of ERK1/2 axis, which was suppressed by EP inhibitor (L-161982) in A549 cells (**Figure 5B** and **Data Sheet S5B**). The aberrant ERK1/2 activation and elevated PTGS2 expression in the resistant A549/DDP cells were also attenuated by either celecoxib or EPs inhibitor (**Figure 5C** and **Data Sheet S5C**). The results implied that PGE2 might strengthen PTGS2 expression through the PGE2-EPs-ERK1/2 positive feedback loop.

### PTGS2 Inhibitor Reversed the Resistance to Cisplatin *In Vivo*

**Figure 6:**
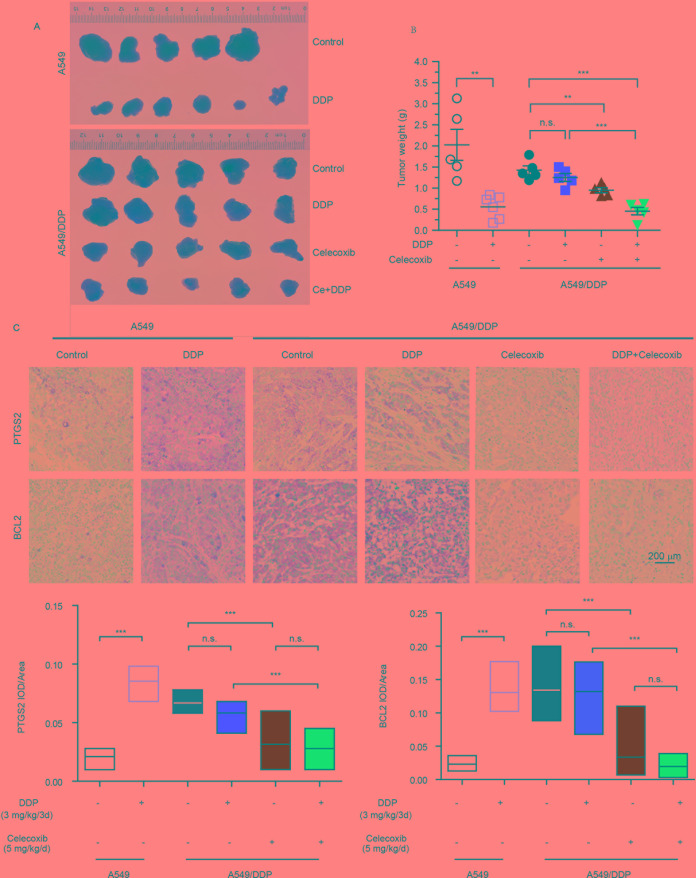
PTGS2 inhibitor reversed the resistance to cisplatin *in vivo*. The A549 (1 × 10^7^ cells) and A5491/DDP cells (5 × 10^6^ cells) were injected into the flank of 5-week-old male BALB/c nude mice, which were randomly distributed into groups. **(A** and **B)** Tumors were weighted after tumor dissection. **(C)** Immunohistochemistry was carried out by probing with anti-PTGS2 mAb (upper) and anti-BCL2 mAb (lower). **P < 0.01, ***P < 0.001, “n.s.” means “no significance.”

The *in vitro* study indicated that PTGS2 induced by cisplatin played an essential role in the chemoresistance of NSCLC cells, suggesting that PTGS2 might serve as a potent target for reversal of chemoresistance. Therefore, we further explored the effects and the underlying mechanisms of PTGS2 inhibitor (celecoxib) on the antitumor activity of cisplatin using the lung cancer xenograft models. The results (**Figures 6A, B** and **Table 2**) showed that administration of cisplatin at a dose of 3 mg/kg once every 3 days significantly inhibited the growth of A549 xenografts, with the tumor inhibition rate up to 72.49%, whereas only 11.94% of inhibition rate was achieved in the resistant A549/DDP xenografts treated with the identical dose of cisplatin. However, co-administration of celecoxib markedly augmented the antitumor effect of cisplatin in the A549/DDP xenografts with the acquisition of cisplatin resistance, increasing the inhibition rate up to 67.98%. Interestingly, moderate suppression effect (33.29% of inhibition rate) was observed in the resistant A549/DDP xenografts treated with the celecoxib alone. No substantial abnormalities caused by administration of celecoxib alone were observed on weights of body, spleen, and kidney (**Figure S2**).

**Table 2:** PTGS2 inhibitor reversed the resistance to cisplatin *in vivo*.

(A)			
Groups	Treatments	Average tumor weight (g)	Inhibition rate (%)
A549	Control	2.028	
	DDP	0.558	72.49
(B)			
Groups	Treatments	Average tumor weight (g)	Inhibition rate (%)
A549/DDP	Control	1.424	
	DDP	1.254	11.94
	Celecoxib	0.950	33.29
	DDP + Celecoxib	0.456	67.98

Further immunohistochemical results indicated that cisplatin-elevated expressions of both PTGS2 and BCL2 were detected in A549 xenografts but not in the resistant A549/DDP models. Compared with treatment of cisplatin, celecoxib or celecoxib combination with cisplatin significantly decreased PTGS2 and BCL2 expressions (**Figure 6C**). Impairment of the PTGS2 activity resulted in decrease of PTGS2 and BCL2 expression and reversal of the resistant NSCLC against the cisplatin, which coincided with the molecular mechanisms as revealed *in vitro*.

### PTGS2 and BCL2 Predicted Poor Survival in NSCLC Patients

**Figure 7:**
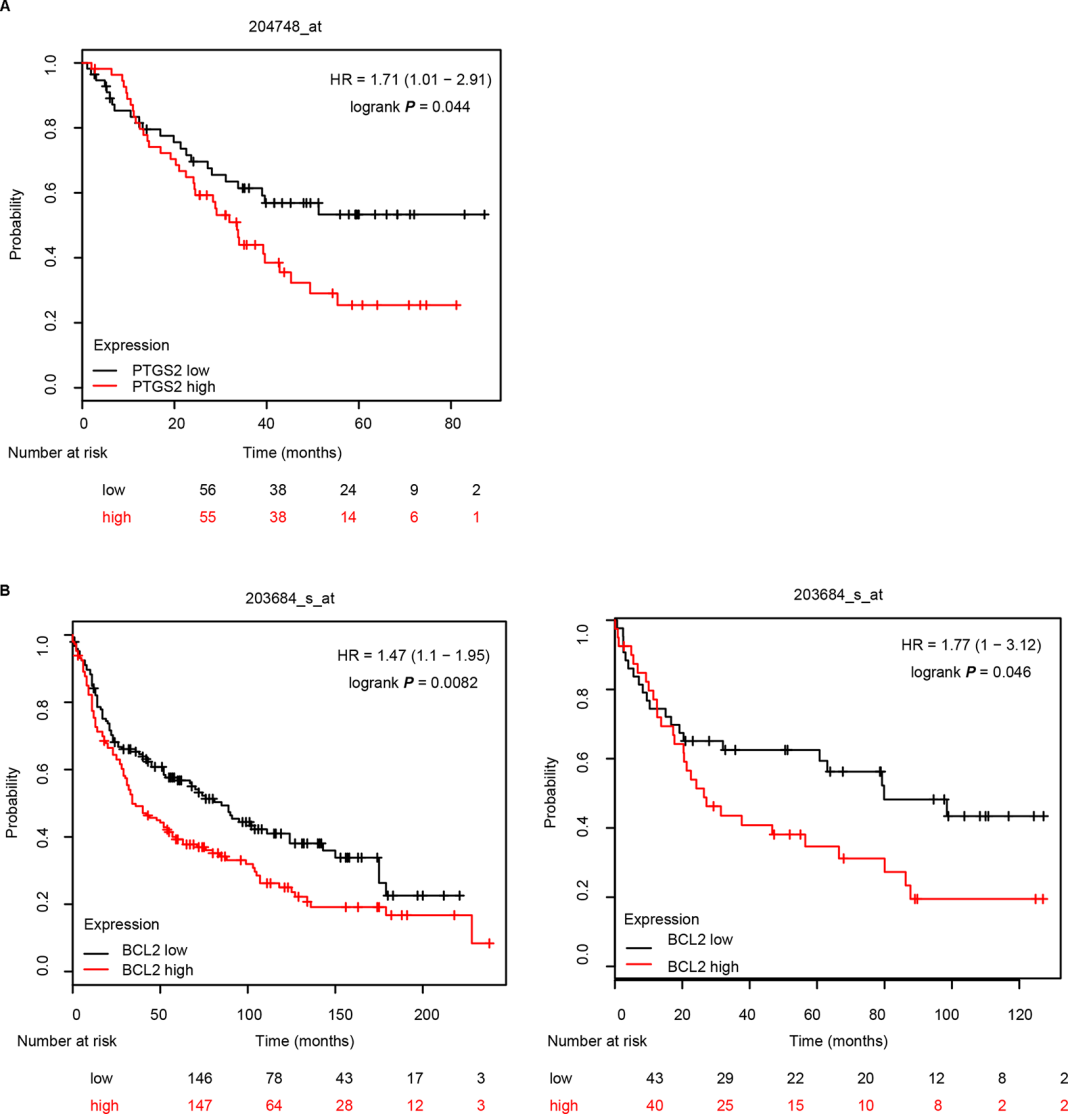
PTGS2 and BCL2 predicted poor survival in NSCLC patients. The relationship between the expression levels of PTGS2 **(A)** and BCL2 **(B)** and the life period was analyzed in the Kaplan–Meier plotter website (http://kmplot.com/lung).

Further analysis of the relationship between the expression levels of PTGS2/BCL2 and the survival rate in the Kaplan–Meier plotter website (http://kmplot.com/lung) indicated that high levels of both PTGS2 (A) and BCL2 (B) were closely associated with poor survival in NSCLC patients (**Figure 7**), implying that PTGS2 and BCL2 abundant in tumor tissues played essential roles in the response of NSCLC therapy. Combined with the above *in vitro* and *in vivo* results, PTGS2 might be employed as an adjunctive therapeutic target for reversal of the chemoresistance in a subset of resistant NSCLC.

## Discussion

Platinum-based chemotherapy is currently the most important standardized regimen for lung cancer (Azzoli et al., 2009; Reck et al., 2014). However, development of the acquired multidrug resistance causes failure of the platinum-based standardized therapy (Chintamani et al., 2004; Li et al., 2014). The clinical trials have shown that lung cancer patients with high levels of PGE2 are insensitive to platinum-based therapy (Altorki et al., 2005). In addition, elevated PGE2 levels were also found in patients who received chemotherapy (Altorki et al., 2005; Edelman et al., 2012). Considering that PGE2 is the major product catalyzed by PTGS2, we speculated that PTGS2 might play important roles in the chemoresistance of platinum-based therapy. As expected, compared with the parental cells, abundant PTGS2 expression and increased PGE2 level were observed in cisplatin-resistant cells. Overexpression of PTGS2 enhanced the chemoresistance, whereas either knocking down of PTGS2 expression or inhibition of its activity restored the sensitivity of NSCLC cells to multiple agents. Moreover, we revealed that cisplatin induced PTGS2 expression through the ROS-ERK1/2-NF-κB signaling axis, and PGE2 might strengthen PTGS2 expression through the PGE2-EPs-ERK1/2 positive feedback loop, which induced multidrug resistance of NSCLC cells *via* mediating the augmentation effects of cisplatin on BCL2 expression and the subsequent impairment of cell apoptosis. It has been reported that PTGS2 potentiated cisplatin resistance of NSCLC by promoting the epithelial–mesenchymal transformation (EMT) through activation of the AKT signaling pathway (Jiang et al., 2019). The novel mechanism provided in this study is different from previous reports that PTGS2 might contribute to drug resistance through promotion of EMT in NSCLC or elevation of P-glycoprotein expression in other tumors (Hosomi et al., 2000; Arunasree et al., 2008; Gu and Chen, 2012).

Given cisplatin induced generation of ROS, which mediated the regulation effects of cisplatin on PTGS2 expression, it was reasonable to propose that scavenging of ROS might inhibit the expression of PTGS2 and BCL2 and thus promote cell apoptosis and improve the sensitivity to cisplatin. However, scavenging of ROS by antioxidant NAC significantly reduced the cisplatin-induced apoptosis (**Figure S3**). Similar results were also obtained in gastric cancer (GC) cells (data unpublished). Therefore, although ROS served as an intermediator for cisplatin inducing resistance, the strategies of targeting ROS to improve the sensitivity of chemotherapy in certain malignancies including NSCLC and GC should be cautious.

Our results identified PTGS2 as an essential mediator implicated in the development of resistance in NSCLC cells exposed to cisplatin, suggesting that PTGS2 might be an effective target for reversal of the acquired resistance. As expected, inhibition of PTGS2 by celecoxib enhanced response of the resistant NSCLC to cisplatin in the xenograft models. Meanwhile, it is noteworthy that moderate inhibition effect was achieved in the resistant NSCLC models treated with the celecoxib alone, whereas little effect of celecoxib administration on the proliferation of resistant cells was observed *in vitro*. It seems that the secreted PGE2 catalyzed by PTGS2 contributes to tumor growth *via* stimulation of the surrounding cells in tumor microenvironment. As celecoxib served as PTGS2 inhibitor and inhibited PGE2 formation, administration of celecoxib might attenuate the effects of PGE2 on tumor microenvironment and indirectly suppressed tumor growth *in vivo*. Further investigations will be required to determine the causes for the discrepant effects of celecoxib *in vitro* and *in vivo*.

PTGS2-derived prostaglandins exert diverse physiological functions, such as maintaining body fluid homeostasis, blood pressure, and organ functions, in an autocrine or a paracrine fashion *via* activation of four subtypes of EP receptors (EP1–4), protecting the organ from excessive functional changes (Breyer and Breyer, 2001; Hao and Breyer, 2008). Inhibition of the enzymatic activity of PTGS2 by celecoxib might reduce the levels of prostaglandins and cause potent side effects. As expected, when combined with cisplatin, celecoxib not only exaggerated the reduced effect of cisplatin on the body weight, but also significantly decreased the kidney weight, which was not influenced when treated with cisplatin alone (**Figure S2**). Notably, we found that administration of celecoxib alone had little effect on the weights of the whole body as well as the spleen and the kidney (**Figure S2**). We speculated that the celecoxib used at a dose of 5 mg/kg once daily could trigger synergistic side effects with the cisplatin, but was not sufficient to influence the detected organ weights by itself. The mechanisms of how the celecoxib synergized with the cisplatin to produce stronger toxicity remained elusive.

In summary, we revealed a novel molecular mechanism of PTGS2 implicated in the chemoresistance of NSCLC cells. Our results demonstrated that cisplatin induced PTGS2 expression through the ROS-ERK1/2-NF-κB signaling axis, and PGE2 might strengthen PTGS2 expression *via* the PGE2-EPs-ERK1/2 positive feedback loop, which induced multidrug resistance of NSCLC cells through up-regulation of BCL2 expression and the subsequent attenuation of cell apoptosis. Inhibition of PTGS2 significantly reversed the chemoresistance in the resistant NSCLC. Our results suggested that PTGS2 might be employed as an adjunctive therapeutic target for improving the response to the therapeutic agents in a subset of cisplatin-resistant NSCLC.

## Data Availability

All datasets generated for this study are included in the manuscript and the **Supplementary Files**. The Western-blot whole images used for the article are displayed in **Data Sheet S1**.

## Ethics Statement

This study was carried out in accordance with the recommendations in the Guidelines for the Care and Use of Laboratory Animals, the Laboratory Animal Ethics Committee of Jinan University. The protocol was approved by the Laboratory Animal Ethics Committee of Jinan University.

## Author Contributions

X-P W designed the experiments and revised the manuscript. X-M L, W L, H W, R-Z L, Y-S H, and L-K C carried out the experiments, analyzed data, and wrote the draft. All authors read and approved the final manuscript.

## Funding

The present study was supported by the National Natural Science Foundation of China (81573334), the Science and Technology Planning Project of Guangdong Province of China (2017A020211029 and 2015A020211017), and the Opening Project of Zhejiang Provincial Top Key Discipline of Pharmaceutical Sciences.

## Conflict of Interest Statement

The authors declare that the research was conducted in the absence of any commercial or financial relationships that could be construed as a potential conflict of interest.

## Abbreviations

PTGS2, prostaglandin-endoperoxide synthase-2; BCL2, B cell lymphoma 2; BAX, BCL2-associated X protein; BCL-XL, B cell lymphoma-extra large; Survivin, baculoviral inhibitor of apoptosis (IAP) repeat-containing 5; PGE2, prostaglandin E2; PGH2, prostaglandin H2; EPs, prostaglandin EP receptors; IC_50_, half maximal inhibitory concentration.
