# Supplementary figures and images for: The Role of Prostaglandin-Endoperoxide Synthase-2 in Chemoresistance of Non-Small Cell Lung Cancer
Source: Front Pharmacol. 2019 Aug 8;10:836. doi: 10.3389/fphar.2019.00836 (PMC6694719; doi:10.3389/fphar.2019.00836)

# Data Sheet 1

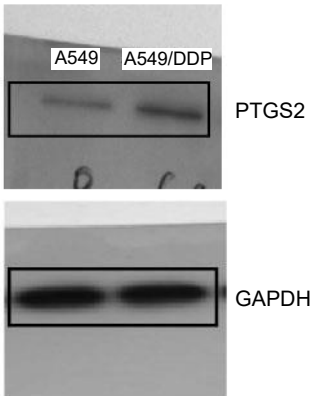

Data Sheet 1. The Western-blot whole image for Figure 1B.

Supplement: Supplementary file 1 [file DataSheet_1.pdf]

Data Sheet 2

(A)

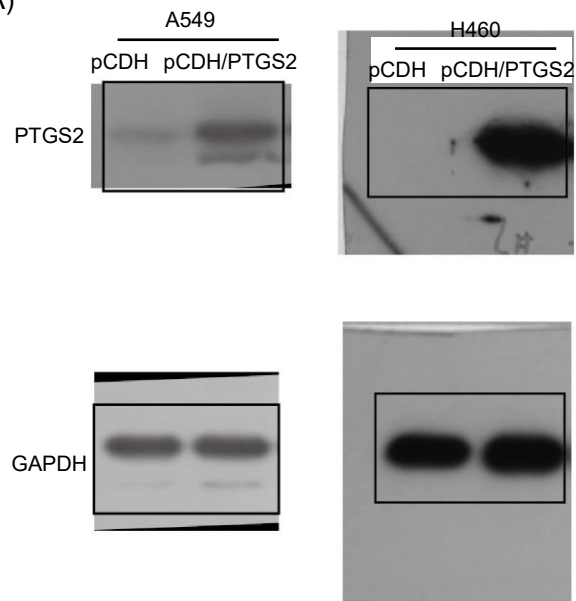

(B)

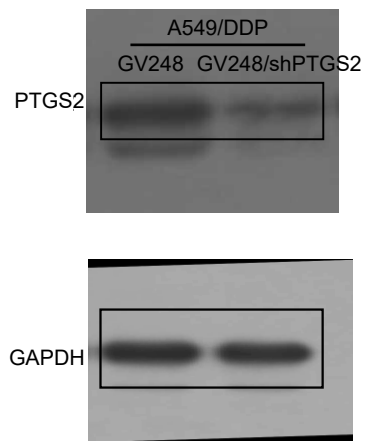

Data Sheet 2. (A-B) The Western-blot whole images for Figure S1(A-B)

Supplement: Supplementary file 2 [file DataSheet_2.pdf]

Data Sheet 3

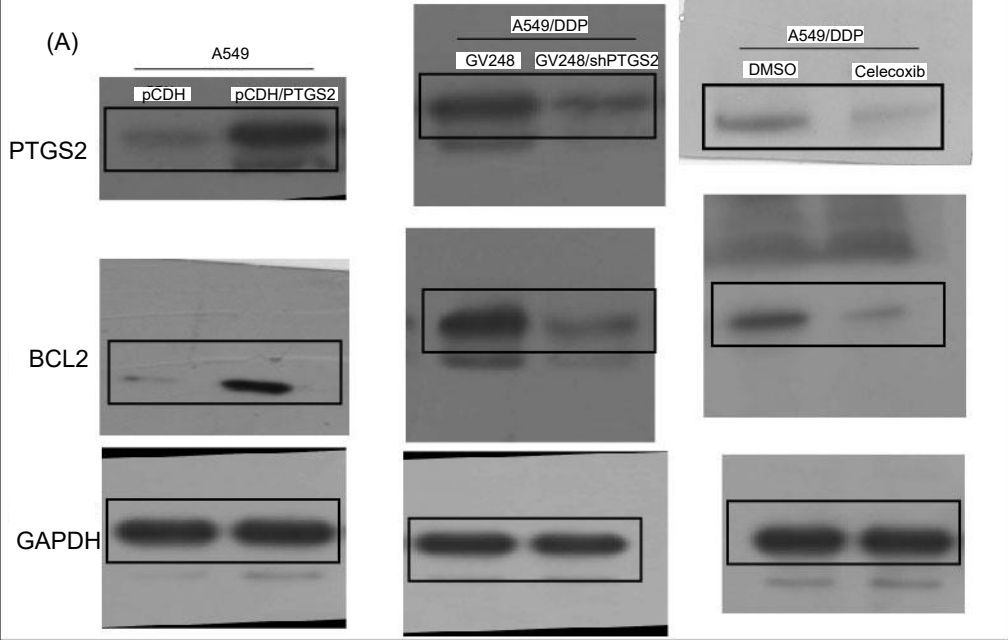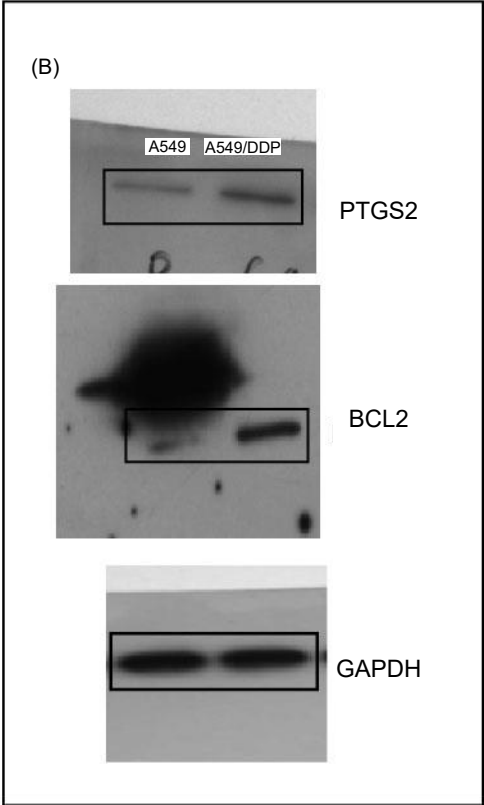

Data Sheet 3. (A-B) The Western-blot whole images for Figure 3B and 3C.

Supplement: Supplementary file 3 [file DataSheet_3.pdf]

Data Sheet 4

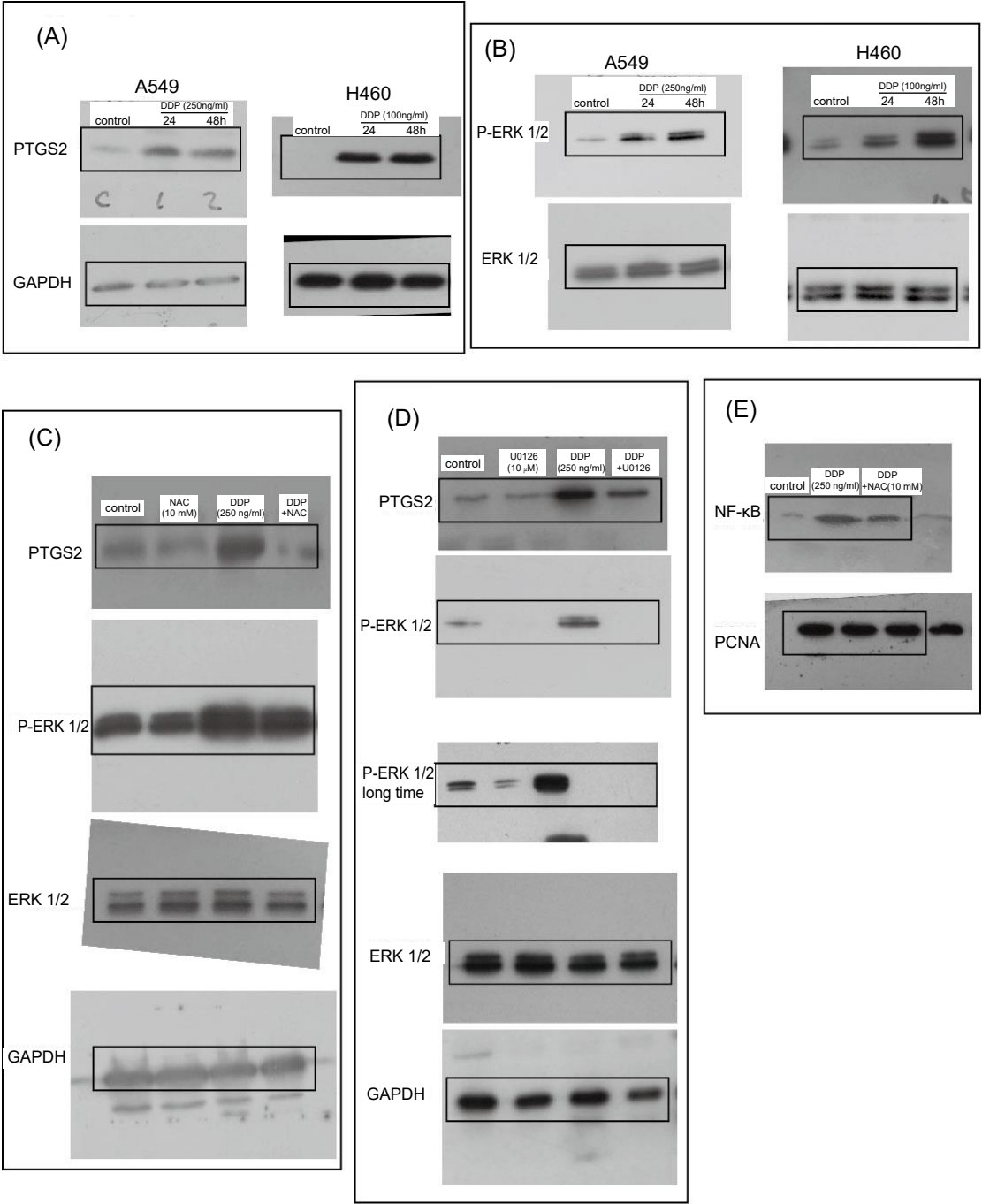

Data Sheet 4. (A-E) The Western-blot whole images for Figure 4(A), 4(C), 4(E), 4(G) and 4(J).

Supplement: Supplementary file 4 [file DataSheet_4.pdf]

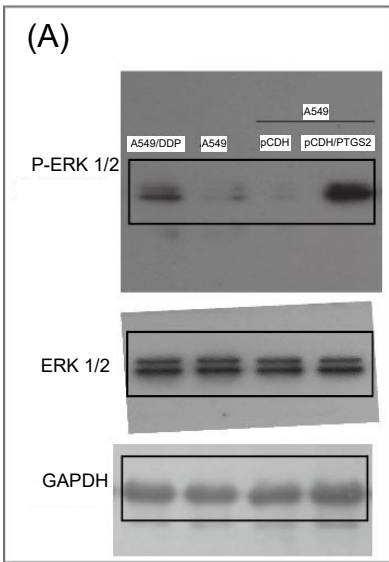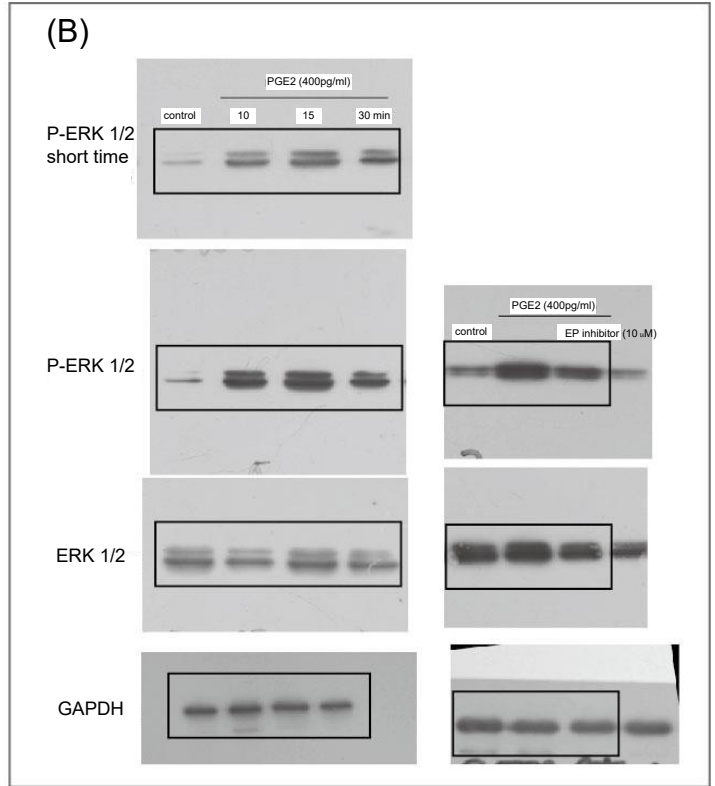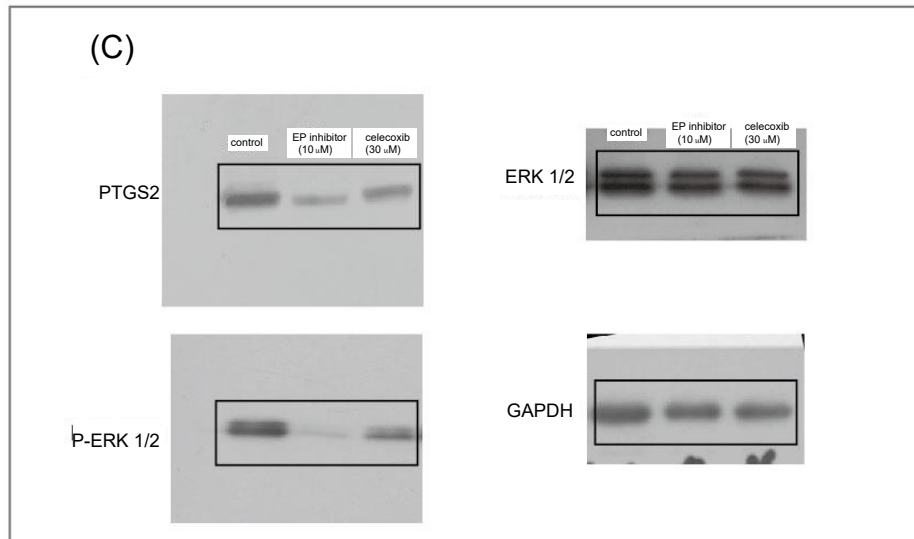

Supplement: Supplementary file 5 [file DataSheet_5.pdf]

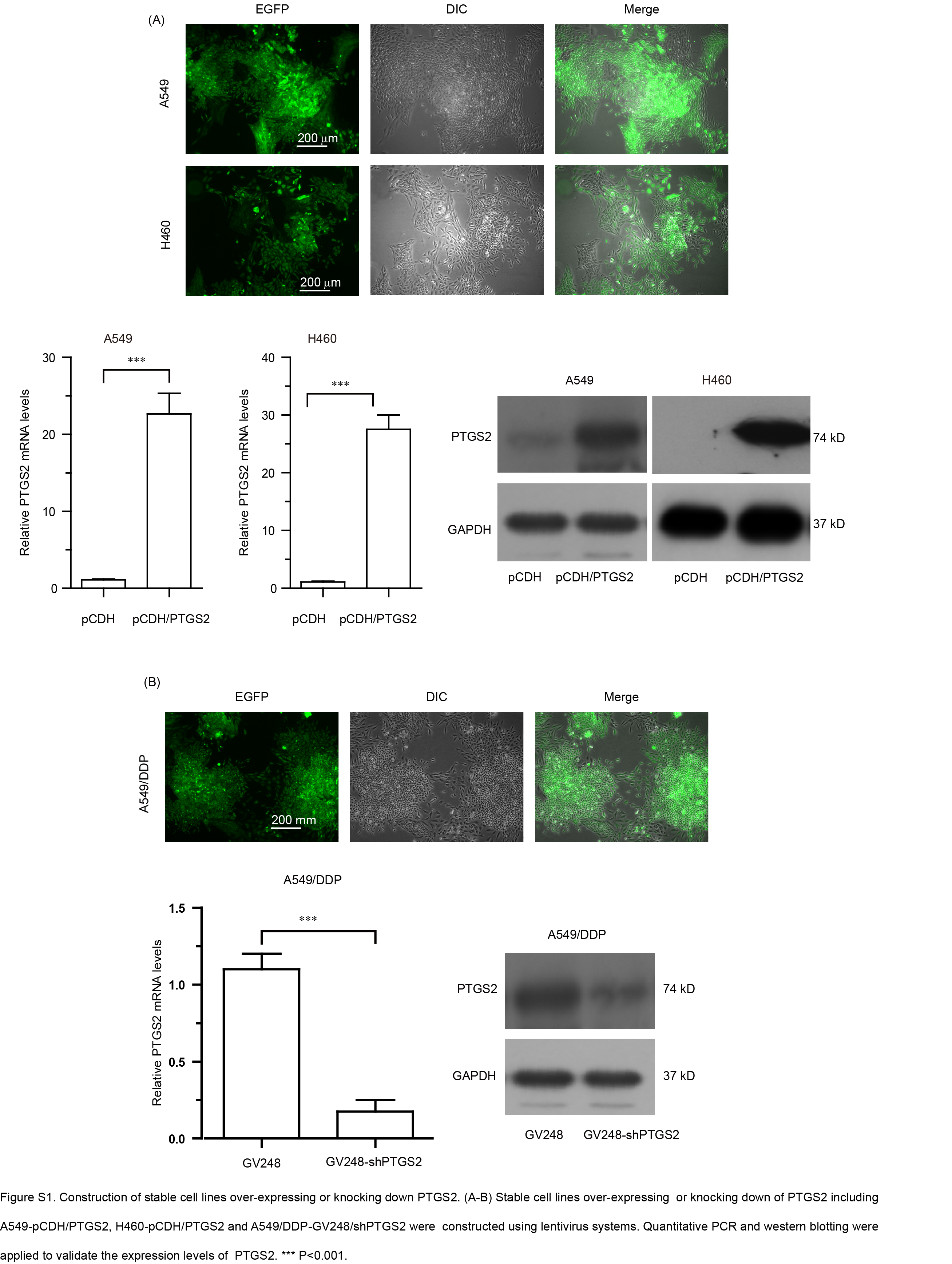

Supplement: Supplementary file 6 [file Image_1.tif]

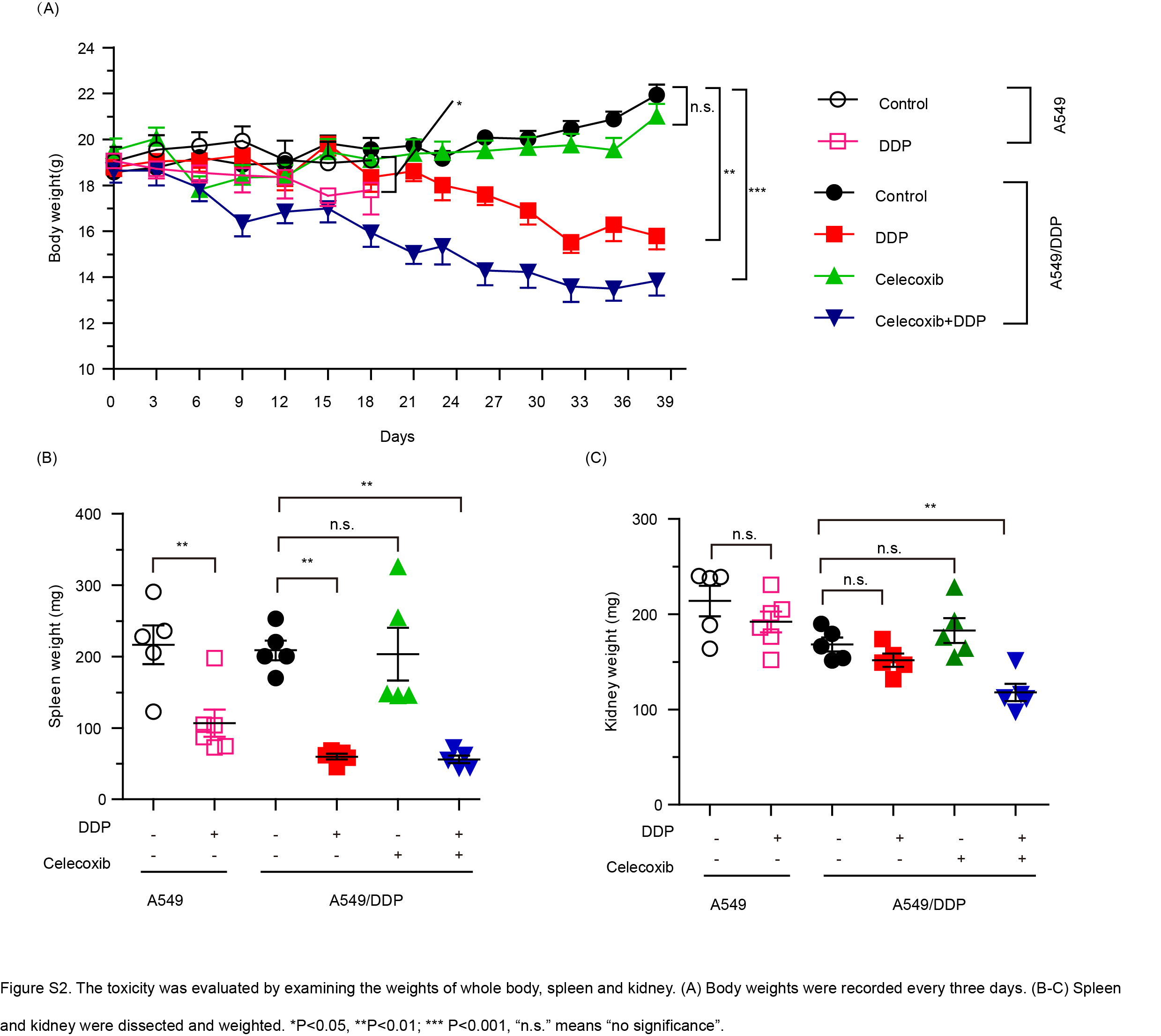

Supplement: Supplementary file 7 [file Image_2.jpg]

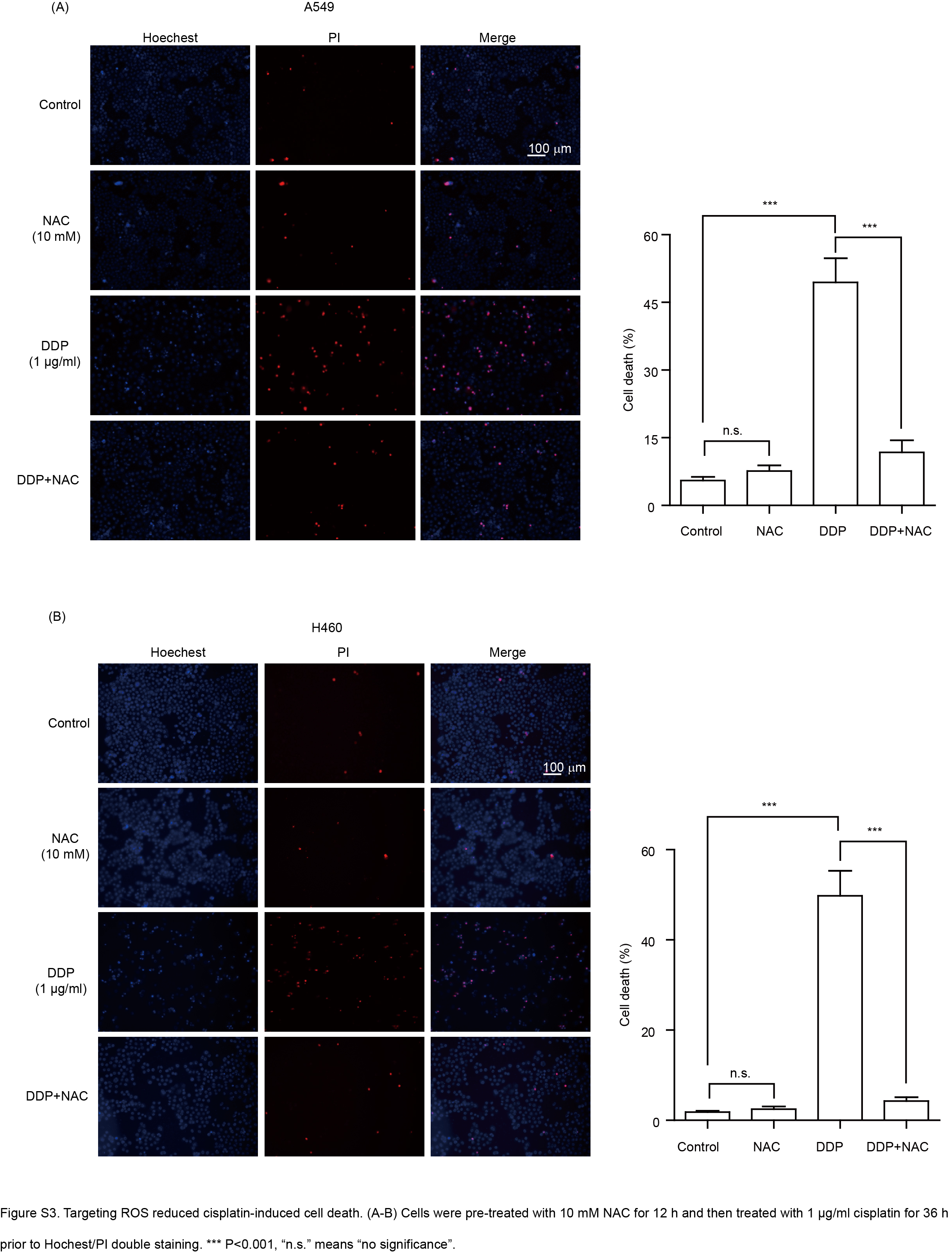

Supplement: Supplementary file 8 [file Image_3.jpg]
