# Supplementary material for: The Role of Prostaglandin-Endoperoxide Synthase-2 in Chemoresistance of Non-Small Cell Lung Cancer
Source: Front Pharmacol. 2019 Aug 8;10:836. doi: 10.3389/fphar.2019.00836 (PMC6694719; doi:10.3389/fphar.2019.00836)
Supplement: Supplementary file 9 [file Table_1.docx]

**Table S1.**

**The primers for quantitative PCR**

| Genes | Forward（5’-3’） | Reverse（5’-3’） |
| --- | --- | --- |
| *BCL-2* | TGCACCTGACGCCCTTCAC | AGACAGCCAGGAGAAATCAAACAG |
| *Survivin* | AACCGGACGAATGCTTTTTA | GATTTGAATCGCGGGACCC |
| *BAX* | AGAGGATGATTGCCGCCGT | CAACCACCCTGGTCTTGGATC |
| *BCL-XL* | GTAAACTGGGGTCGCATTGT | TGCTGCATTGTTCCCATAGA |
| *PTGS2* | ACCGCAAACGCTTTATGCTG | AAAGATGGCATCTGGCCGA |
| *GAPDH* | CCCACTCCTCCACCTTTGAC | TGTGCTGTAGGAAGCTCA |

**The primers for constructing pCDH/PTGS2 plasmid**

Forward（5’-3’）: CCGGAATTCGCCGTGGCCGCCGCCGCGATG

Reverse（5’-3’）: GCGCGGATCCAGACTTCTACAGTTCAGTCGAACG

**The following oligonucleotides were used to create the shPTGS2 construct**

Forward（5’-3’）:

ccggtGATTATGTGCAACACTTGATtcaagagATCAAGTGTTGCACATAATCttttttG

Reverse（5’-3’）:

AATTCaaaaaaGATTATGTGCAACACTTGATctcttgaATCAAGTGTTGCACATAATCa
