# Supplementary material for: The Role of Prostaglandin-Endoperoxide Synthase-2 in Chemoresistance of Non-Small Cell Lung Cancer
Source: Front Pharmacol. 2019 Aug 8;10:836. doi: 10.3389/fphar.2019.00836 (PMC6694719; doi:10.3389/fphar.2019.00836)
Supplement: Supplementary file 10 [file Table_2.docx]

**Table S2. A549/DDP cells displayed higher resistance to DDP, ADM and GEM than A549 cells.**

| Cells | IC_50_ | | |
| --- | --- | --- | --- |
|  | DDP(μg/mL) | ADM(μg/mL) | GEM(μg/mL) |
| A549 | 1.2±0.02 | 1.1±0.11 | 0.5±0.13 |
| A549/DDP | 4.1±0.33 | 4.5±0.45 | 8.1±0.56 |
